# A comparison of routine blood culture methods and multiplex quantitative PCR for detecting pathogens in simulated polymicrobial blood cultures

**DOI:** 10.1099/jmm.0.002155

**Published:** 2026-04-10

**Authors:** Mariam Doualeh, Christopher Mullally, Martin Thorsen, Edward Raby, Edward Litton, Soraya Leedham, Matthew Payne, Andrew Currie

**Affiliations:** 1 School of Medical, Molecular, and Forensic Sciences, Murdoch University, Perth, WA, Australia; 2 Precision Medicine Centre, Murdoch University, Perth, WA, Australia; 3 Wesfarmers Centre for Vaccines and Infectious Diseases, The Kids Research Institute Australia, Perth, WA, Australia; 4 Microbiology Department, Australian Clinical Labs, Osborne Park, WA, Australia; 5 Infectious Diseases and Microbiology Department, Fiona Stanley and Fremantle Hospital Group, Murdoch, WA, Australia; 6 Microbiology Department, PathWest Laboratory Medicine, Murdoch, WA, Australia; 7 Intensive Care Unit, Fiona Stanley Hospital, South Metropolitan Health Service, Perth, WA, Australia; 8 Division of Obstetrics and Gynaecology, School of Medicine, The University of Western Australia, Perth, WA, Australia

**Keywords:** blood culture, infection, multiplex quantitative PCR, polymicrobial bacteraemia, sepsis

## Abstract

**Introduction.** Polymicrobial bacteraemia, the simultaneous presence of multiple bacterial species in the bloodstream, complicates diagnosis and treatment and is linked to poorer patient outcomes. Accurate detection is essential for effective clinical management.

**Gap Statement.** Despite being the diagnostic gold standard, conventional blood culture may fail to detect all pathogens in polymicrobial infections, particularly when species differ in growth rate or abundance. The relative performance of culture versus molecular methods under these conditions remains poorly characterized.

**Aim.** To compare the performance of conventional blood culture and quantitative PCR (qPCR) for detecting pathogens in simulated polymicrobial blood cultures containing fast- and slow-growing bacteria at varying ratios and concentrations.

**Methodology.** Four clinically relevant polymicrobial mixtures were prepared using seven bacterial species. Human blood was spiked with bacterial suspensions at varying ratios and inoculated into BACTEC blood culture bottles. After incubation, samples were analysed using standard culture techniques and an in-house multiplex qPCR assay.

**Results.** Routine culture detected both organisms in only 42% of samples, while qPCR identified both pathogens in 83%. Differences in bacterial growth rates significantly influenced culture outcomes, with slower-growing or less abundant species frequently missed. Notably, *Staphylococcus aureus* was not detected when co-cultured with *Escherichia coli* at ratios of 1:1 to 25:1, and only visible on Gram stain when *S. aureus* was initially 50 times more abundant.

**Conclusion.** These findings highlight a key limitation of conventional methods in detecting polymicrobial infections and underscore the need for more sensitive diagnostics. qPCR offers improved detection, particularly when organism abundance and growth rates vary. Incorporating molecular tools alongside routine culture may enhance diagnostic accuracy and provide a more complete understanding of polymicrobial bacteraemia.

## Introduction

Sepsis, a life-threatening organ dysfunction typically caused by a dysregulated host response to infection, remains a major global health burden [1], with an annual global incidence estimated at ~49 million cases, resulting in 11 million deaths worldwide – a mortality rate of around 22% [2]. Early and accurate detection of bacteraemia, the presence of bacteria in the bloodstream, is crucial for effective clinical management and improved patient outcomes in sepsis, as it guides the timely adjustment of empirical antibiotic therapy – the cornerstone of treatment [1].

Polymicrobial bacteraemia, defined as the presence of more than one bacterial species in the bloodstream, presents unique diagnostic and therapeutic challenges in sepsis. These infections account for ~6–15% of all bacteraemia cases and have been associated with worse patient outcomes, including prolonged hospital stays and a reported 2–3 times higher mortality rate [3,5]. Polymicrobial bacteraemia is most frequently observed in individuals with risk factors such as compromised immune systems, chronic diseases (e.g. diabetes and cancer), recent surgical procedures or the presence of invasive devices such as central venous catheters [3,7]. Consequently, common pathogens implicated in polymicrobial bacteraemia include a range of both Gram-positive and Gram-negative bacterial species [5,7]. Gut bacteria, particularly *Enterococcus* species and members of the *Enterobacteriaceae* family, are frequently implicated due to the gastrointestinal tract being the most common source of infection [3,4, 7,10]. Other notable pathogens include *Pseudomonas aeruginosa* and *Acinetobacter baumannii*, while in neonates, infections often involve skin organisms, particularly coagulase-negative staphylococci [3,6, 8]. Clinically, certain pathogen pairings may occur more frequently depending on the infection source. For example, *Enterobacterales* with enterococci or anaerobic Gram-negative bacteria in intra-abdominal infections [11,12], *Enterobacterales* with *Staphylococcus aureus* in severe burn infections [13] and *S. pneumoniae* with Gram-negative organisms in patients with severe underlying conditions [14] have been reported.

Traditional blood culture methods, considered the gold standard for detecting bacteraemia, involve directly inoculating patient blood samples at point of collection into specialist culture bottles containing a defined aerobic or anaerobic atmosphere and nutrient-rich media with antibiotic-neutralizing substrates, which are then incubated in automated instruments that continuously monitor for of bacterial growth [15]. Once growth is flagged, a Gram stain from a culture smear provides preliminary identification, and then, samples are sub-cultured onto solid agar plates for further species-level identification and antibiotic susceptibility testing [15]. However, in polymicrobial scenarios, Gram staining to guide antibiotic therapy adjustment can be problematic; a rapidly growing organism may overshadow the presence of other slower-growing pathogens on the Gram stain, potentially leading to incomplete or inaccurate treatment decisions [6]. It can take several days to yield definitive results, during which time patients often receive broad-spectrum empirical antibiotics [15]. Additionally, the method often exhibits reduced sensitivity in the presence of low bacterial loads, which can occur due to factors such as prior antibiotic use or the inherently low volume of blood typically collected from vulnerable patients such as neonatal and paediatric patients [16,17].

In cases of polymicrobial bacteraemia, pathogens may present at different concentrations, with one pathogen potentially dominating the other/s, even before culture is initiated. Furthermore, pathogens may exhibit significantly different growth rates in the same culture medium [18,19]. Such disparities could then be amplified during the automated blood culture process, accentuating the presence of a more dominant pathogen. As a result, less prevalent or slower-growing pathogens may be missed in diagnostic outcomes, compromising the accuracy of diagnosis and the efficacy of appropriate antibiotic selection. Beyond differences in growth rates, interactions between co-cultured bacteria such as nutrient competition, metabolic by-products or inhibitory compounds may also modify the growth behaviour of individual species and influence their detectability in polymicrobial cultures. Understanding these dynamics and interactions between pathogen species during culture is crucial for improving diagnostic methods and ensuring that all pathogens are appropriately treated.

The limitations of conventional blood culture systems in detecting polymicrobial bacteraemia have not been comprehensively explored [20]. Previous research has focused only on fungal–bacterial co-infections, leaving a knowledge gap regarding the impact on mixed bacterial infections [18,19]. Moreover, there is a lack of data regarding the performance of culture systems in detecting polymicrobial bacteraemia in samples with low bacterial loads, a critical concern in neonatal patients, as well as some paediatric and adult patients from whom obtaining sufficient blood samples is challenging.

Recent advances in molecular diagnostic techniques, such as multiplex quantitative PCR (qPCR), next-generation sequencing and metagenomics, have improved pathogen detection by overcoming some of the limitations of traditional blood culture methods, particularly in terms of sensitivity and turnaround time [21]. While not yet widely adopted in routine clinical laboratories, these molecular approaches allow for direct pathogen detection from blood culture bottles or whole blood, bypassing the need for sub-culturing on solid media [22]. However, it remains unclear whether these methods outperform traditional culture techniques in the detection of polymicrobial infections, especially where multiple pathogens are present in varying concentrations.

To address these gaps, we employed a sensitive, in-house multiplex qPCR assay alongside routine culture methods to analyse mock polymicrobial blood cultures. By simulating various bacterial inoculation ratios and using organisms with differing growth rates using commonly detected sepsis pathogens, we evaluated the detection rates of both methods and identified potential limitations of routine blood culture in accurately diagnosing polymicrobial bacteraemia. This research is particularly relevant for clinical laboratories and healthcare providers seeking to improve diagnostic accuracy and patient outcomes in the context of bloodstream infections.

## Methods

### Bacterial isolates

Seven bacterial strains isolated from clinical blood cultures were included in this study: *Enterococcus faecalis*, *Escherichia coli*, *S. aureus*, *Enterobacter cloacae*, *Klebsiella pneumoniae*, *Bacteroides fragilis* and *Streptococcus pneumoniae*. These organisms are among the most clinically significant pathogens in sepsis and polymicrobial bloodstream infections across adult, paediatric and local neonatal populations, with reported distributions varying by infection source and patient cohort [3,9, 23,25].

To simulate polymicrobial infections, four combinations were examined: *E. cloacae+E. faecalis*, *E. coli+S. aureus*, *E. coli+S. pneumoniae* and *K. pneumoniae+B. fragilis*. These pairings reflect clinically documented co-infection patterns reported in immunocompromised patients, intra-abdominal infections and burn-associated infections [11,14] and encompass both Gram-positive and Gram-negative species, aerobic and anaerobic bacteria and pathogens with differing growth kinetics (approximate doubling times of organisms listed in Table 1).

**Table 1.** Doubling times of pathogens used in the study

Organism	Doubling time under optimal laboratory conditions	Growth classification
*E. coli*	~20 min [55]	Fast
*S. aureus*	30–40 min [56,57]	Fast
*S. pneumoniae*	40–55 min [58]	Moderate
*E. cloacae*	20–50 min [59,61]	Fast-moderate
*E. faecalis*	~40 min [62]	Moderate
*K. pneumoniae*	30–40 min [63,64]	Fast
*B. fragilis*	90–120 min [65]	Slow

### Bacterial culture

For each strain, a single colony from a 5% horse blood agar (HBA; Thermo Fisher Scientific) plate was transferred into sterile culture broth. *E. faecalis*, *E. coli*, *S. aureus*, *E. cloacae* and *K. pneumoniae* were cultured in Luria–Bertani (LB) broth (Oxoid), while *B. fragilis* and *S. pneumoniae* were cultured in Brain–Heart Infusion (BHI) broth (Sigma-Aldrich) supplemented with l-cysteine and hemin. Liquid cultures were then incubated overnight in a 37 °C shaker incubator (220 r.p.m.), except for *B. fragilis* and *S. pneumoniae*, which were incubated at 37 °C without shaking. A small aliquot of the overnight culture was transferred to 5 ml of sterile broth to achieve an OD at 600 nm (OD_600_) of ~0.05, restarting the culture. These secondary cultures were then incubated under the same respective conditions as the primary cultures. Duplicate OD_600_ measurements were taken at 30 min intervals (60 min intervals for *S. pneumoniae* and *B. fragilis* due to slower growth) until the end of the logarithmic phase to determine the time required to reach the mid-log phase.

At each time point, bacterial suspensions were spot-plated onto HBA (Thermo Fisher Scientific) and incubated at 37 °C under organism-specific conditions: aerobic for *S. aureus*, *E. coli*, *E. cloacae*, *E. faecalis* and *K. pneumoniae*; 5% CO₂ for *S. pneumoniae*; and anaerobic for *B. fragilis*. Plates were incubated overnight or longer if needed. Colony counts were conducted the following day to determine concentrations (c.f.u./ml). A standard curve was generated by plotting OD_600_ against c.f.u./ml, enabling the calculation of equations correlating OD_600_ values with c.f.u./ml measurements.

### Inoculum preparation for mock cultures

The liquid cultures of each bacterial strain were grown to the mid-logarithmic phase and then diluted to achieve final concentrations of either 100 c.f.u. ml^−1^ or 1,000 c.f.u. ml^−1^ through serial tenfold dilutions. The concentrations were verified by spot plating on HBA plates (Thermo Fisher Scientific). These diluted suspensions served as the working stocks for inoculating the mock blood cultures.

### Preparation of mock adult and neonatal/paediatric blood cultures

Each pathogen combination was evaluated across five conditions: monoculture of organism A, monoculture of organism B, a 1 : 1 combination of both organisms, a 1 : 10 combination of A and B and a 10 : 1 combination of A and B. Testing included three combinations representative of paediatric cases and one simulating adult conditions.

The ‘paediatric’ model was designed to identify challenges in detecting polymicrobial infections at low bacterial loads. Neonatal and paediatric bacteraemia typically occurs at very low concentrations (~1–50 c.f.u. ml^−1^), and clinical blood draws in this group can yield <1 ml of blood [17,26]. Therefore, the initial inoculum was set at 10 c.f.u., using pathogen combinations commonly implicated in sepsis for this group (*E. coli*, *S. aureus*, *E. faecalis*, *E. cloacae* and *S. pneumoniae*). BACTEC Peds Plus blood culture bottles without fastidious organism supplement (BD Diagnostics) were inoculated with 100 µl of pre-mixed inoculum suspension and 500 µl of heparinized donor blood. The three pathogen combinations were *E. cloacae+E. faecalis*, *E. coli+S. aureus* and *E. coli+S. pneumoniae*.

To evaluate whether increased blood volume and higher starting bacterial load affected detection, an ‘adult’ model was incorporated. Adult bacteraemia also typically presents at low concentrations, although levels may rise to 10³–10⁴ c.f.u. ml^−1^ [27]. Accordingly, an initial inoculum of 100 c.f.u. was selected to represent this wider range of bacterial loads. Sets of BACTEC Plus Aerobic/F and BACTEC Lytic/10 Anaerobic/F blood culture bottles (BD Diagnostics) were inoculated with 8 ml of donor blood (recommended minimum) and 100 µl of bacterial suspension. The pathogens selected for this model (*K. pneumoniae* and *B. fragilis*) were chosen for their clinical relevance in adult sepsis.

Negative controls comprising 100 µl of sterile LB or BHI broth and donor blood (500 µl for paediatric model and 8 ml for the adult model) were also included. All bottles were loaded onto a BACTEC FX instrument (BD Diagnostics) within 30 min of inoculation.

### Routine blood culture analysis

Microscopy and culture analyses were conducted by independent technical staff at the microbiology department of an accredited pathology laboratory (Australian Clinical Labs, Osborne Park, Western Australia). Once a positive BACTEC signal was flagged by the automated instrument, samples underwent Gram staining, followed by sub-culturing onto HBA (Thermo Fisher Scientific). In cases where Gram-negative bacilli (GNB) were identified in the Gram stain, samples were additionally sub-cultured onto MacConkey Agar (MCA; Thermo Fisher Scientific). Samples exhibiting Gram-positive cocci (GPC) were sub-cultured on Colistin Nalidixic Acid (CNA) agar (Thermo Fisher Scientific), while samples showing a mixture of Gram types were sub-cultured on both MCA and CNA. Plates were incubated at 37 °C, with anaerobic incubation performed when required, using BD GasPak containers and pouches (BD Diagnostics). Plates were observed for growth the following day and again after 36–48 h if no growth was observed. Blood culture bottles were stored at 4 °C (up to 24 h) before bacterial load quantification and qPCR analysis. Short-term refrigeration at 4 °C has been shown not to adversely affect organism viability [28].

### Bacterial load quantification

The final bacterial concentration of each pathogen in a combination, following a positive flag on the automated system, was determined by colony counting. This was achieved by spot plating on the selective and differential solid media listed in Table 2. Briefly, serial tenfold dilutions of the blood culture were prepared, and 20 µl of each dilution was spotted onto agar plates in triplicate. Following incubation (under species-specific conditions), colonies were counted for the dilution that yielded 10–20 colonies. The average number of colonies was then multiplied by the dilution factor to determine the original bacterial concentration.

**Table 2.** Selective and differential agar used for colony counting

Combination	Media used
*E. coli+S. aureus*	MCA (Thermo Fisher Scientific) for *E. coli*+CNA (Thermo Fisher Scientific) for *S. aureus*
*E. cloacae+E. faecalis*	MCA (Thermo Fisher Scientific) for *E. cloacae*+CNA (Thermo Fisher Scientific) for *E. faecalis*
*E. coli+S. pneumoniae*	MCA (Thermo Fisher Scientific) for *E. coli*+CNA (Thermo Fisher Scientific) for *S. pneumoniae*
*K. pneumoniae+B. fragilis*	MCA (Thermo Fisher Scientific) for *K. pneumoniae*+Bacteroides Bile Esculin Agar for *B. fragilis*

### Bacterial DNA extraction and qPCR

Following routine processing, a 1.8 ml aliquot was aspirated from blood culture bottles, and DNA was extracted using the MagAttract Microbial DNA Kit (QIAGEN) on the KingFisher Duo automated extraction platform following the manufacturer’s protocol (Thermo Fisher Scientific). PCR reactions were performed using the QuantStudio 6 real-time PCR system, employing an established in-house multiplex qPCR sepsis panel previously detailed (manuscript under review). The panel covers the following pathogens: *E. coli*, *Serratia marcescens*, *E. cloacae*, *E. faecalis*, *Klebsiella* spp., *P. aeruginosa*, *S. aureus*, coagulase-negative staphylococci, *Enterococcus faecium*, *Streptococcus pyogenes* and *B. fragilis*. Each reaction comprised a total volume of 20 µl, consisting of 4 µl PerfeCTa multiplex qPCR ToughMix (Quantabio), 300 nM forward primer, 300 nM reverse primer, 150 nM probe, 3 µl template and nuclease-free water (Invitrogen, Thermo Fisher Scientific). Cycling conditions included an initial denaturation step at 95 °C for 5 min, followed by 40 cycles of 95 °C for 20 s and 60 °C for 20 s (data acquisition). Data were analysed using QuantStudio software (v1.6.1).

### Threshold investigation in *E. coli* and *S. aureus* co-culture

Initial Gram stain analysis of the *E. coli* and *S. aureus* combination showed no detectable *S. aureus* at any of the tested ratios. To investigate the threshold concentration of *S. aureus* necessary for its detection in the Gram stain when co-cultured with *E. coli*, supplementary mock blood cultures with varying ratios of *E. coli* to *S. aureus* (1 : 10, 1 : 25, 1 : 50 and 1 : 100) were established. Microscopy, culture, colony counting and qPCR were performed following the methods detailed above, with one variation: CNA sub-cultures (which would normally only be added if a Gram stain indicates the presence of Gram-positive organisms) were prepared for each combination, irrespective of the Gram stain results.

## Results

### Routine diagnostic blood culture vs PCR

All spiked bottles yielded a positive signal on the automated blood culture system except for the aerobic *B. fragilis* monoculture. Both the routine and PCR methods detected at least one pathogen in all combinations (Table 3). The routine method correctly identified both pathogens, confirmed by accurate Gram staining and growth following routine sub-culture, in 5 out of 12 combinations (41.7%), whereas PCR detected both pathogens in 10 out of 12 combinations (83%). The two cases missed by PCR both involved the *B. fragilis* target (*B. fragilis+K. pneumoniae* at a 1 : 1 ratio and *B. fragilis+K. pneumoniae* with a tenfold excess of *K. pneumoniae*) and were also undetected by routine culture. Although both aerobic and anaerobic blood culture bottles were inoculated for the *K. pneumoniae+B. fragilis* combination, only the anaerobic bottles were analysed, as *B. fragilis*, a strict anaerobe, was not expected to grow in aerobic bottles, while *K. pneumoniae* exhibited comparable growth under both conditions.

**Table 3.** Microscopy, culture and PCR detection of common sepsis pathogens from blood culture. Comparison of routine methods and qPCR for pathogen detection across various combinations and ratios of polymicrobial blood cultures. The table highlights differences in time to positivity (TTP), Gram stain results, subculture morphology and final pathogen identification by each method

Combination	Ratio	Pathogen/s inoculated (c.f.u.)	Mean TTP	Gram stain	Subculture morphology			Final pathogen detection	
					HBA	MCA	CNA	Routine	PCR
**Combination 1** **(paediatric)**	Monoculture	*E. coli* (10)	8 h 37 min (+/− 24 min)	GNB	Single	Single	na	EC	EC
	Monoculture	*S. aureus* (10)	13 h 52 min (+/− 7 min)	GPC	Single	na	Single	SA	SA
	1 : 1	*E. coli* (5)+*S. aureus* (5)	8 h 55 min	GNB	Single	Single	na	EC	EC+SA
	1 : 10	*E. coli* (1)+*S. aureus* (9)	9 h 19 min (+/− 8 min)	GNB	Single	Single	na	EC	EC+SA
	10 : 1	*E. coli* (9)+*S. aureus* (1)	8 h 50 min (+/− 7 min)	GNB	Single	Single	na	EC	EC+SA
**Combination 2** **(paediatric)**	Monoculture	*E. cloacae* (10)	9 h 26 min (+/− 20 min)	GNB	Single	Single	na	ECl	ECl
	Monoculture	*E. faecalis* (10)	9 h 55 min (+/− 5 min)	GPC	Single	na	Single	EF	EF
	1 : 1	*E. cloacae* (5)+*E. faecalis* (5)	9 h 46 min (+/− 14 min)	GNB+GPC	Double	Single	Single	ECl+EF	ECl+EF
	1 : 10	*E. cloacae* (1)+*E. faecalis* (9)	10 h 6 min (+/− 6 min)	GNB+GPC	Double	Single	Single	ECl+EF	ECl+EF
	10 : 1	*E. cloacae* (9)+*E. faecalis* (1)	9 h 12 min (+/− 13 min)	GNB+GPC	Double	Single	Single	ECl+EF	ECl+EF
**Combination 3** **(paediatric)**	Monoculture	*E. coli* (10)	8 h 49 min (+/− 5 min)	GNB	Single	Single	na	EC	EC
	Monoculture	*S. pneumoniae* (10)	15 h 48 min (+/− 30 min)	GPC	Single	na	Single	SP	SP
	1 : 1	*E. coli* (5)+*S. pneumoniae* (5)	9 h 43 min (+/− 23 min)	GNB+scant GPC	Single	Single	Single	EC+SP	EC+SP
	1 : 10	*E. coli* (1)+*S. pneumoniae* (9)	10 h 34 min (+/− 14 min)	GNB+scant GPC	Single	Single	Single	EC+SP	EC+SP
	10 : 1	*E. coli* (9)+*S. pneumoniae* (1)	9 h 13 min	GNB	Single	Single	na	EC	EC+SP
**Combination 4** **(adult)**	Monoculture	*K. pneumoniae* (100)	7 h 39 min (+/− 26 min)	GNB	Single	Single	na	KP	KP
	Monoculture	*B. fragilis* (100)	26 h 43 min	GNB	Single	Single	na	BF	BF
	1 : 1	*K. pneumoniae* (50)+*B. fragilis* (50)	7 h 50 min (+/− 20 min)	GNB	Single	Single	na	KP	KP
	1 : 10	*K. pneumoniae* (10)+*B. fragilis* (90)	8 h 38 min (+/− 31 min)	GNB	Single	Single	na	KP	KP+BF
	10 : 1	*K. pneumoniae* (90)+*B. fragilis* (10)	7 h 33 min (+/− 18 min)	GNB	Single	Single	na	KP	KP

BF, *B. fragilis*; EC, *E. coli*; ECl, *E. cloacae*; EF, *E. faecalis*; KP, *K. pneumoniae*; SA, *S. aureus*; SP, *S. pneumoniae*.

Differences in blood volumes and culture media types did not impact detection rates in these simulations. The paediatric model used 500 µl blood with BACTEC Peds Plus bottles, while the adult model used 8 ml blood in BACTEC Plus Aerobic/F and Lytic/10 Anaerobic/F bottles. Despite differences in total blood volume and initial inoculum (10 c.f.u. in paediatric versus 100 c.f.u. in adult), detection outcomes were primarily influenced by pathogen ratios and growth dynamics rather than these factors. Both methods successfully detected both pathogens in the 1 : 1 ratio for the *E. cloacae+E. faecalis* and *E. coli+S. pneumoniae* combinations, but not in the *E. coli+S. aureus* and *B. fragilis+K. pneumoniae* combinations. The disparities in detection were especially pronounced when evaluating the 1 : 10 and 10 : 1 ratios, where the routine method failed to detect both pathogens in all cases, except for the *E. cloacae+E. faecalis* (1 : 10 and 10 : 1) and *E. coli+S. pneumoniae* (10 : 1) combinations.

### Final bacterial concentrations

**Fig. 1.**
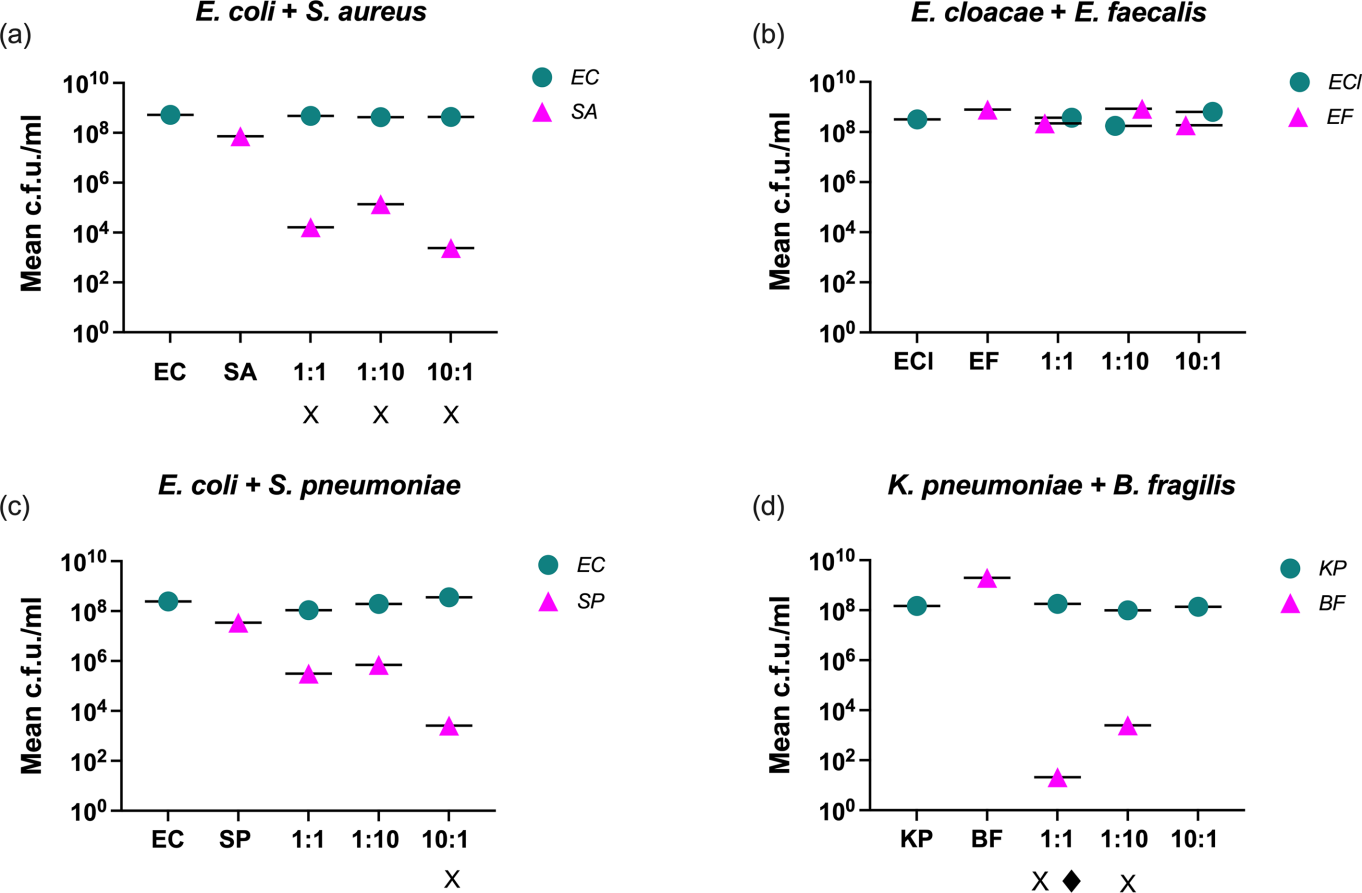
Final bacterial concentrations (c.f.u./ml) following BACTEC incubation. Detection outcomes by routine blood culture and qPCR are indicated below the x-axis, with missed detections marked as X (less abundant pathogen missed by routine culture) and ♦ (less abundant pathogen missed by qPCR). EC, *E. coli*; SA, *S. aureus*; ECl, *E. cloacae*; EF, *E. faecalis*; SP, *S. pneumoniae*; KP, *K. pneumoniae*; BF, *B. fragilis*. 1 : 1=equal starting concentrations of both pathogens. 1 : 10=10× *S*. *aureus* starting concentration (panel a), 10× *E. faecalis* starting concentration (panel b), 10× *S*. *pneumoniae* starting concentration (panel c) and 10× *B. fragilis* starting concentration (panel d). 10 : 1=10× *E. coli* starting concentration (panel a), 10× *E. cloacae* starting concentration (panel b), 10× *E. coli* starting concentration (panel c) and 10× *K*. *pneumoniae* starting concentration (panel d). In the 10 : 1 *K*. *pneumoniae+B. fragilis* combination, *B. fragilis* failed to grow on spot plates, resulting in no data.

Fig. 1 depicts the final bacterial concentrations (c.f.u./ml) of positive blood cultures after automated incubation, determined by spot plating on selective media for both target organisms – a step that is not part of the routine diagnostic workflow. The automated system required bacterial growth to exceed 10⁷ c.f.u. ml^−1^ to trigger a positive flag, as evidenced by the monomicrobial culture results. Among these, the lowest load was detected for *S. pneumoniae* at 3.47×10^7^ c.f.u. ml^−1^ and the highest for *B. fragilis* at 1.97×10^9^ c.f.u. ml^−1^.

In the *E. cloacae+E. faecalis* combination, both pathogens reached similar final concentrations (≥10⁸ c.f.u. m^−1^) across all ratios, leading to consistent detection by both methods (Fig. 1b). In contrast, the remaining combinations displayed notable log differences between co-cultured species, which impacted detection outcomes. For the *E. coli+S. aureus* combination, *E. coli* consistently dominated, reaching concentrations exceeding 10⁸ c.f.u. ml^−1^, while *S. aureus* was detected at final concentrations three logs lower, even when inoculated at a 10× higher initial ratio (Fig. 1a). For the *E. coli+S. pneumoniae* combination, both pathogens were detected in all mixtures except the 10 : 1 combination (10× the *E. coli*); here, additional non-routine sub-culture identified *S. pneumoniae* at a final concentration of 2.59×10^3^ c.f.u. ml^−1^, more than three logs lower than *E. coli* (Fig. 1c).

In the *K. pneumoniae+B. fragilis* combination, *K. pneumoniae* was consistently detected by both methods across all ratios, reaching >10⁸ c.f.u. ml^−1^, whereas *B. fragilis* was not detected by routine methods (Fig. 1d). Plate counts revealed final *B. fragilis* concentrations of 21 c.f.u. ml^−1^ in the 1 : 1 ratio and 2.5×10³ c.f.u. ml^−1^ in the 10 : 1 ratio (10× *B. fragilis*). No *B. fragilis* growth was observed on spot plates from the 10 : 1 mixture (10× *K. pneumoniae*).

### Supplementary *E. coli*+*S. aureus* cultures

Given that the routine method failed to detect *S. aureus* in all tested ratios in combination with *E. coli*, additional mock blood cultures of *E. coli* and *S. aureus* combinations were established. These aimed to explore the minimal threshold concentration of *S. aureus* required for its identification in the Gram stain when co-cultured with *E. coli*. *E. coli* was consistently detected by both methods across all combinations (Table 4). While *S. aureus* was identifiable via PCR in all combinations, it was not detected in the Gram stain at the 1 : 10 and 1 : 25 ratios. It only became visible when its initial concentration was increased to 50× and 100× that of *E. coli*, with a final concentration of at least 1.37×10^6^ c.f.u. ml^−1^ (Table 4). Growth on blood agar gave pure *E. coli* colonies in all combinations except the 1 : 100 ratio, which showed mixed colonies. *S. aureus* growth was, however, observed on CNA plates for all combinations.

**Table 4.** Detection of *S. aureus* in co-culture with *E. coli* at various ratios. *S. aureus* was missed by microscopy and culture at lower ratios (1 : 10 and 1 : 25) but detected by qPCR in all cases. Gram staining only identified *S. aureus* when its final concentration exceeded 1.37×10⁶ c.f.u. ml^−1^ (1 : 50 and 1 : 100 ratios)

Combination (*E. coli* : *S. aureus*)	Final c.f.u./ml		Gram stain	Sub-culture			PCR
	*E. coli*	*S. aureus*		HBA	MCA	CNA	
1 : 10	3.46×10^8^	1.71×10^5^	GNB	Pure	Pure	Pure	EC+SA
1 : 25	3.56×10^8^	3.41×10^5^	GNB	Pure	Pure	Pure	EC+SA
1 : 50	2.48×10^8^	1.37×10^6^	GNB+GPC	Pure	Pure	Pure	EC+SA
1 : 100	2.22×10^8^	7.82×10^6^	GNB+GPC	Mixed	Pure	Pure	EC+SA

EC, *E. coli*; SA, *S. aureus*.

## Discussion

The present study aimed to investigate potential limitations of a standard blood culture diagnostic workflow in accurately detecting pathogens in polymicrobial bacteraemia cases, when variations exist in bacterial loads and/or growth rates. The investigation stems from recognized shortcomings of traditional blood culture methods, notably their reduced sensitivity in detecting low bacterial loads [29], and the lack of data available regarding performance in detecting polymicrobial bacteraemia [20]. By employing a more sensitive qPCR test, alongside routine culture analysis of mock polymicrobial blood cultures, we uncovered a notable discordance in detection rates. While both methods identified at least one pathogen in all cases, routine diagnostic workflows frequently failed to detect the second pathogen when the starting ratios and/or growth rates of the organisms varied considerably. While previous studies have examined the limitations of blood culture systems using mock fungal–bacterial combinations, to our knowledge, none have directly compared multiplex qPCR with the gold standard culture method to assess these limitations in mock blood cultures containing mixed bacterial species.

Analysis of the TTP results for individual pathogens in monoculture revealed significant disparities in their growth rates. Only the combination of *E. cloacae* and *E. faecalis* exhibited similar TTP values (<1 h difference). Consequently, this was the only combination wherein both methods consistently detected both pathogens when co-cultured across all tested ratios. Conversely, clear disparities in growth rates were observed in the other combinations, evidenced by significant TTP differences in monocultures: over 5 h for *E. coli+S. aureus*, more than 7 h for *E. coli+S. pneumoniae* and exceeding 19 h for *K. pneumoniae+B. fragilis*. These findings highlight a limitation of automated blood culture systems, which monitor bacterial growth by measuring changes in parameters, such as CO_2_ levels, and use proprietary algorithms to flag a bottle as positive once a detectable level is reached. According to our findings and previous reports, the bacterial threshold for detection is between 10^7^ and 10^9^ c.f.u. ml^−1^ [30]. If one pathogen in a polymicrobial culture grows significantly faster, it may reach this detection threshold while slower-growing pathogens remain undetectable upon microscopy and routine sub-culture.

This scenario was exemplified in our study. Initial diagnostic procedures involve preparing Gram stains directly from positive blood culture bottles, using a single drop to prepare the smear. The limit of detection for Gram stains from liquid cultures is generally estimated to be around 10^5^ c.f.u. ml^−1^, though variability exists [31]. For instance, incerebrospinal fluid samples, detection rates have been reported to increase from 25% for ≤10^3^ c.f.u. ml^−1^ to over 97% for >10^5^ c.f.u. ml^−1^ [32]. In our study, Gram stain failed to detect pathogens in co-culture when the less abundant organism was at concentrations below ~10^5^ c.f.u. ml^−1^. The Gram stain’s limit of detection may be higher in polymicrobial cultures than in monocultures due to the small volume of culture used for the smear, which can decrease the likelihood of detecting less abundant pathogens. This issue is further amplified in polymicrobial cultures with a dominant pathogen, as it may overshadow less prevalent organisms, particularly if the bottles are not adequately mixed before testing.

Gram stains hold dual significance in the diagnostic workflow. Firstly, they guide clinicians in determining whether adjustments are necessary in empirical antimicrobial therapy before final antimicrobial susceptibility results are available, which can take over 24 h [15]. Failure to detect a pathogen in the Gram stain of a polymicrobial culture could lead clinicians to erroneously narrow antimicrobial coverage. This was documented in a neonatal study where preliminary Gram stain results from clinical samples containing both Gram-positive and Gram-negative pathogens indicated only one type in 16% of the polymicrobial episodes, delaying adequate therapy adjustments until complete identification and susceptibility testing were available [6].

Secondly, Gram stains guide laboratory staff in selecting selective and differential agar for routine sub-culture to enhance the likelihood of isolating single colonies of all pathogens present. In our study, while the diagnostic laboratory adjusted its approach based on Gram stain results, we utilized selective and differential media to quantify the concentration of the two pathogens in combination, irrespective of Gram stain results. Both pathogens showed quantifiable growth in all combinations except for *B. fragilis* when *K. pneumoniae* was spiked at a tenfold greater concentration. The recovery of most pathogens on these additional media, including those missed by the routine method, underscores the masking effect of the dominant species. Undetected pathogens were potentially present on the blood agar sub-culture used by the diagnostic laboratory, however, likely only in the primary and secondary inoculum, thereby precluding their identification.

This observation was reinforced in the supplementary *S. aureus+E. coli* testing, where the diagnostic laboratory was instructed to set up CNA plates regardless of the Gram stain results. Here, *S. aureus* grew on CNA plates in all tested ratios even when not picked up on the Gram stain and blood agar. Using Gram-specific selective and differential media, regardless of Gram stain results, could reduce delays in adjusting antimicrobial therapy by enabling earlier detection of missed pathogens. This approach is particularly valuable, as blood culture bottles are typically not re-incubated once flagged as positive by automated systems. Consequently, suppressed organisms may only be detected if isolated from a subsequent blood draw – possibly at a later stage of infection. Prompt detection ensures timely intervention and more effective antimicrobial treatment [15].

Variations in growth rates provide one explanation for missed pathogen detection, but they do not fully account for all our findings. Notably, in the case of the *S. aureus+E. coli* combination, *S. aureus* remained undetected in all scenarios, even when initially present at concentrations tenfold that of *E. coli*, whereas *S. pneumoniae* was consistently detected in co-culture with *E. coli*, except when the initial *E. coli* concentration exceeded that of *S. pneumoniae* by tenfold. This outcome was unexpected, given the superior growth of *S. aureus* compared to *S. pneumoniae* (evidenced by shorter TTP in monoculture), which typically poses greater challenges in laboratory cultivation. Subsequent supplementary testing revealed that *S. aureus* remained undetected by the routine method in co-culture with *E. coli* until its concentration was increased fiftyfold relative to *E. coli*. These observations suggest that additional factors may influence detection in this specific combination. Possible explanations include the presence of interspecies interactions, potentially involving nutrient competition (e.g. for carbon sources such as glucose) or growth suppression via metabolic by-products (e.g. lactic acid) or toxins [33,36]. This effect appeared less evident in the *E. coli+S. pneumoniae* cultures, suggesting potential species-specific dynamics. These interactions may also be strain-dependent; for example, *E. coli* strains have previously been shown to kill multiple *S. aureus* strains through the production of the genotoxin colibactin without compromising their own viability, although this phenotype is not universal [36].

Manufacturers offer various media for blood culture systems catering to aerobic, anaerobic, fastidious and fungal organisms. It is plausible that the composition of the BACTEC Peds Plus media (optimized for low blood volumes and fastidious organisms) does not favour *S. aureus* in certain co-culture scenarios. The importance of using optimal blood culture bottles has been highlighted in mixed fungal–bacterial cultures, where the failure to employ specific mycosis blood culture bottles significantly reduces the ability to detect fungi in polymicrobial cultures with bacteria [18,19]. While this is expected due to differences in growth and nutrient requirements between fungi and bacteria, similar considerations may apply to specific bacterial combinations. Further investigation into the optimal nutrient requirements and inter-species interactions in bacterial co-cultures is warranted, although the proprietary status of blood culture media components presents challenges.

Blood culture bias disproportionately affected the sensitivity of routine methods to detect both pathogens compared to qPCR. Overall, our qPCR demonstrated superior performance in detecting both pathogens. However, there were two instances where qPCR failed: *B. fragilis* in the 1 : 1 and 10 : 1 ratios with *K. pneumoniae* (10× *K*. *pneumoniae*). Our previous work shows that in BACTEC Peds Plus bottles, this qPCR assay reliably detects *B. fragilis* in monoculture within 4 h, starting from a bacterial load of 50 c.f.u., and within 5 h starting from 5 c.f.u., under BACTEC-simulated incubation conditions (manuscript under review). In this study, the failure to detect *B. fragilis* after 7.5–8.5 h of BACTEC incubation when combined with *K. pneumoniae* in 1 : 1 (50 c.f.u. each) and 10 : 1 (90 c.f.u. *K. pneumoniae* and 10 c.f.u. *B. fragilis*) suggests that some aspect of the mixed-culture dynamics may have impaired its detection. Although a significant difference in growth rates is the most plausible explanation, additional mechanisms are potentially involved. *K. pneumoniae*, for instance, efficiently sequesters key nutrients such as iron via siderophores, potentially limiting *B. fragilis* growth [37]. Furthermore, certain *K. pneumoniae* strains produce bacteriocins or other antimicrobial peptides that can inhibit the growth of competing microbes, which may have contributed to the suppression observed in this study [38].

These findings underscore how growth biases can mask the presence of clinically relevant pathogens, even when sensitive molecular methods like qPCR are employed. This has important implications for diagnostic test validation, highlighting the need to routinely include polymicrobial cultures – an aspect that is often overlooked. This is particularly relevant as diagnostic laboratories increasingly explore matrix-assisted laser desorption/ionization-time of flight (MALDI-TOF) testing directly from blood culture bottles to reduce turnaround times. However, direct MALDI-TOF testing presents significant limitations when applied to polymicrobial samples. For example, one study found that direct MALDI-TOF correctly identified all pathogens in only 2.1% of polymicrobial cultures [39].

Overall, our PCR exhibited superior performance over the routine blood culture method for detecting both pathogens. PCR’s high sensitivity allows for the detection of even low levels of pathogens that other methods might miss. Additionally, its multiplexing capabilities enable the simultaneous detection of multiple pathogens in a single test, making it especially effective for detecting polymicrobial infections [21]. Studies have shown that qPCR can identify polymicrobial bacteraemia cases missed by routine culture methods, leading to timely and accurate adjustments in antimicrobial therapy and improving patient outcomes [40,42]. However, although multiplex blood-culture PCR panels can reduce time to targeted therapy and have demonstrated cost-effectiveness in some clinical contexts, their broader implementation is limited by the higher per-test and capital costs compared with alternative methods like MALDI-TOF MS [43]. For this reason, the adoption of multiplex qPCR, potentially combined with viability PCR approaches such aspropidium monoazide-qPCR to minimize false-positive detection of non-viable organisms [44], should be evaluated carefully to ensure that the clinical benefits justify the financial and workflow implications for the intended patient population.

Another key limitation of qPCR is its dependence on the pathogens included in the multiplex panel, which creates unique challenges for validation and verification of PCR as a new clinical laboratory method when using traditional culture as the gold standard. Even with the most comprehensive panels, such as the BioFire FilmArray Blood Culture Identification 2, which targets 33 pathogens, false negatives can occur if the pathogen in the sample is not included in the panel [45,47]. One approach to partially overcome this limitation is to use 16S rRNA gene-based Gram-specific PCR in parallel, targeting conserved regions within bacterial genomes [48,49]. While this method does not offer species-level identification, it can differentiate between Gram-positive and Gram-negative bacteria, enabling the detection of less common pathogens not included in standard multiplex panels and helping to guide empirical antimicrobial therapy [48,49]. However, a key consideration is the potential for non-specific amplification due to mis-priming, including with human DNA – a possibility particularly relevant in adult samples, where larger blood volumes and associated higher white blood cell counts in blood culture bottles may increase this likelihood.

Despite its limitations, qPCR can be integrated with other diagnostic approaches to provide a more comprehensive understanding of bloodstream infections. Augmenting PCR with less targeted molecular approaches like metagenomics could further enhance detection and understanding by providing insights into diverse pathogens, including bacteria, fungi and viruses, along with their antimicrobial resistance profiles and functional potential [50,51]. Although metagenomics is unlikely to become a first-line diagnostic tool in the near future, due to challenges such as high costs, complex data interpretation and lengthy turnaround times, it is poised to play an increasingly complementary role, especially in addressing complex or atypical infections.

This study had several limitations. Firstly, bacteria cultured under laboratory conditions may behave differently from those in clinical settings, where factors such as growth phase and environmental stresses can influence their behaviour. While we aimed to mimic clinical scenarios as closely as possible, the controlled conditions of our study do not fully replicate the complex environment found in real bloodstream infections, namely where the host’s immune response plays a key role. Specifically, our study utilized bacteria in mid-logarithmic phase, which does not accurately reflect the heterogeneous growth phases present in clinical samples, which can affect their detection and behaviour in diagnostic tests [52]. Additionally, we only tested combinations of two pathogens using a single clinical strain to represent each, whereas complex polymicrobial infections can involve more than three pathogens, potentially reducing test sensitivity [39]. Furthermore, our evaluation was limited to the BACTEC system (BD Diagnostics), whereas the BacT/ALERT system (bioMérieux) is also widely used in clinical laboratories. While overall differences in performance between the two systems have been comparable, studies suggest that each system might favour the growth of certain pathogens depending on the bottles used and whether antibiotics have been administered [53,54]. Differences have also been noted in detecting fungi in polymicrobial samples, suggesting that similar comparisons may be necessary for polymicrobial bacteraemia [19].

Despite these limitations, our findings indicate that blood culture sensitivity is compromised in polymicrobial samples, potentially leading to an underreporting of polymicrobial bacteraemia. Addressing this issue requires dedicated efforts and resources to validate these findings in clinical samples. There is a lack of information on polymicrobial bacteraemia and sepsis, particularly in paediatric and neonatal populations, highlighting a critical gap in understanding that must be addressed. The clinical implications also remain unclear; most importantly, it is uncertain whether antimicrobials administered in polymicrobial infections inadvertently treat undetected pathogens or if these pathogens persist and contribute to treatment failure or recurrent infections. Additionally, undetected pathogens may later be identified as a new, unrelated infection, which could lead to misinterpretation. Further clinical research is essential to elucidate these complexities and inform more effective diagnostic and therapeutic strategies for the management of polymicrobial bacteraemia and sepsis.

## Abbreviations

BHI: Brain–Heart Infusion
CNA: Colistin Nalidixic Acid
GNB: Gram-negative bacilli
GPC: Gram-positive cocci
HBA: horse blood agar
LB: Luria–Bertani
MALDI-TOF: matrix-assisted laser desorption/ionization-time of flight
MCA: MacConkey Agar
OD_600_: OD at 600 nm
qPCR: quantitative PCR
TTP: time to positivity
